# Dehydration is a strong predictor of long‐term prognosis of thrombolysed patients with acute ischemic stroke

**DOI:** 10.1002/brb3.849

**Published:** 2017-10-18

**Authors:** Sha‐Sha Li, Ming‐Ming Yin, Zhong‐He Zhou, Hui‐Sheng Chen

**Affiliations:** ^1^ Jinzhou Medical University JinZhou China; ^2^ Department of Neurology General Hospital of Shenyang Military Region ShenYang China

**Keywords:** blood urea nitrogen/creatinine, dehydration, ischemic stroke, prognosis, thrombolysis, urine specific gravity

## Abstract

**Background and Purpose:**

Dehydration was found to be involved in the poor prognosis of patients with acute ischemic stroke. It is unclear whether dehydration status before onset is related with prognosis of thrombolysed patients with acute ischemic stroke. If it is the case, quickly hydrating may improve the prognosis. The present study was designed to explore the issue.

**Methods:**

Eligible 294 patients with acute ischemic stroke after thrombolysis were enrolled in the present study according to inclusion/exclusion criteria. According to the modified Rankin scale (mRS) 90 days post stroke, the patients were divided into two groups: mRS 0–2 (*n *= 191) and mRS 3–6 (*n *= 103). In the present study, BUN/Cr ≥ 15 combined with USG > 1.010 or either of them were chosen as dehydration marker. Clinical data were analyzed between two groups. Univariate and multivariate statistical analyses were carried out.

**Results:**

Age, fibrinogen, blood glucose, BUN/Cr, NIHSS score at admission, the systolic blood pressure (SBP) before thrombolysis, dehydration status (BUN/Cr ≥ 15 plus USG > 1.010), hyperlipidemia, USG and D‐dimer on admission day, and TOAST classification showed significant difference between two groups (*p *< .05). Further stratification analysis showed that BUN/Cr ≥ 15, NIHSS ≥ 6, blood glucose ≥8, and SBP > 150 were markedly associated with poor outcome (mRS 3–6, *p* < .05). After adjusting for age, fibrinogen, USG, D‐dimer, dehydration status, NIHSS, blood glucose, SBP, hyperlipidemia, and BUN/Cr at admission, multivariate logistic regression showed that dehydration status, higher NIHSS, higher blood glucose, and higher SBP at admission were independent risk factors for predicting the long‐term poor prognosis of thrombolysed patients.

**Conclusions:**

The present findings suggest that BUN/Cr ≥ 15 combined with USG > 1.010 as a marker of dehydration status was an independent risk factor for long‐term poor prognosis of thrombolysed patients with acute ischemic stroke.

## 1 INTRODUCTION

Some parameters, including plasma osmolality, the blood urea nitrogen (BUN) to creatinine (Cr) ratio (BUN/Cr), and urine specific gravity (USG), have been used to estimate dehydration status and outcomes in patients with ischemic stroke (Lee et al., 2011; O'Neill et al., 1992; Schrock, Glasenapp, & Drogell, 2012). Among these parameters, BUN/Cr and USG are widely used to assess dehydration status, especially in patients with normal renal function. Several studies have shown that BUN/Cr ratio as a marker of dehydration status was an independent predictor of early neurological deterioration (END) and poor outcomes 30 days post stroke among patients who had suffered acute ischemic stroke (Aronson et al., 2008; Lin et al., 2011; Vullo‐Navich et al., 1998; Weinberg et al., 1994). Some findings suggest that USG as a marker of dehydration status may be associated with stroke in evolution in patients with acute ischemic stroke (Lin et al., 2011; Schrock et al., 2012). In these studies (Akimoto et al., 2011; Bhatia et al., 2015; Lin et al., 2011; Schrock et al., 2012), BUN/Cr ≥ 15 or USG > 1.010 was often used as a maker of dehydration status, but the conclusion about the role of dehydration status in ischemic stroke was controversial. USG > 1.030 has also been reported as a marker in only one study with subjects in an ultralow‐humidity environment (Su et al., 2006). Dehydration is common among stroke subjects and is associated with poor outcome (Chang et al., 2016). Hypoperfusion, renal impairment, and hypercoagulability associated with dehydration status may contribute to the development of ischemic stroke (Bhalla et al., 2000; Jauch et al., 2013; Kelly et al., 2004). It is not clear as to which parameters is best to investigate the relationship between dehydration status and ischemic stroke.

Thrombolytic therapy is an effective and safe treatment for acute ischemic stroke. Many factors such as older age, diabetes mellitus, a history of atrial fibrillation, and initial stroke severity have been demonstrated to be related to the poor outcome of thrombolysed patients (Stefanovic Budimkic et al., 2017). However, the role of dehydration in the prognosis of acute ischemic stroke patients with thrombolytic treatment has not yet been investigated. We argue that if dehydration status before thrombolysis is involved in the poor outcome of the patients, quickly hydrating may increase the recanalization rate with resulting improved outcome. Thus, this study was designed to determine whether dehydration is involved in the prognosis of thrombolysed patients.

## 2 MATERIALS AND METHODS

### 2.1 Patients

To investigate dehydration and the prognosis of patients with acute cerebral infarction of thrombolysis. From our prospective thrombolysis database of consecutive patients with acute ischemic stroke who only received rt‐PA (0.9 mg/kg, maximum 90 mg; manufacturer: Boehringer Ingelheim; production batch number: 505483) from December 2014 to September 2016, thrombolysed patients with acute ischemic stroke were recruited to the present study. Inclusion criterion must be as follows: acute ischemic stroke confirmed by brain CT or MRI scan; rt‐PA treatment could be started within 4.5 hr of onset. Patients were excluded if: (1) the time between the onset of neurological symptoms and rt‐PA treatment was more than 4.5 hr, (2) they had evidence of hemorrhagic stroke assessed by CT, (3) they required endovascular intervention, (4) they had the underlying disease including chronic renal failure (Cr > 2 mg/dl) or liver cirrhosis, and (5) they required diuretics. According to the modified Rankin scale (mRS) 90 days post stroke, the patients were divided into two groups: mRS 0–2 group and mRS 3–6 group. The study was approved by the Ethics Committee of General Hospital of Shenyang Military Region.

### 2.2 Procedure

The following data were collected from the participants using a standardized data collection form: (1) basic clinical characteristics—age, sex, history of diabetes, history of cerebral infarction, myocardial infarction, hypertension, hyperlipidemia, blood pressure at admission, and smoking habits; (2) laboratory investigations—BUN, white blood cell count, red blood cell, Cr, BUN/Cr ratio, blood glucose at admission, D‐dimer (qualitative assay), USG, and fibrinogen; (3) neurological function—NIHSS at admission and mRS 90 days after onset. The etiology of stroke was classified according to TOAST classification (Ay, 2010). Urine and blood samples were obtained and tested on the admission day. In the present study, BUN/Cr ≥ 15, USG > 1.010, or BUN/Cr ≥ 15 combined with USG > 1.010 (dehydration status) was chosen as dehydration marker.

### 2.3 Statistical analysis

The data were compiled and analyzed using standard statistical methods and relevant conclusions were drawn using a computer‐based software SPSS version 24.0. Continuous data were expressed as mean ± standard deviation (SD) and were compared using Student's *t* test for normally distributed variables and Mann–Whitney *U* test for nonparametric data. Categorical data were expressed as frequencies and percentages, and were compared using chi‐square test or Fisher's exact test, wherever applicable. Multivariable analysis was done by logistic regression to determine factors related to long‐term prognosis. *p *<* *.05 was considered significant.

## 3 RESULTS

**Figure 1:**
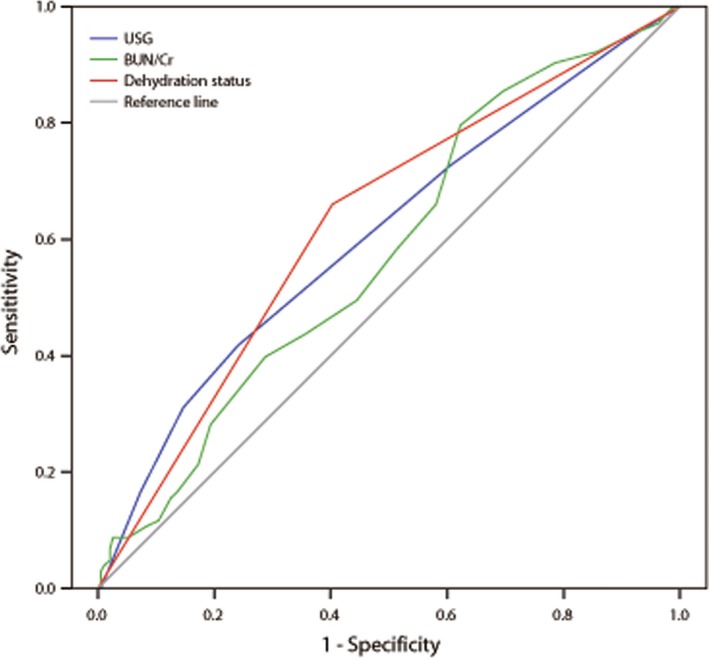
BUN/Cr ≥ 15, USG > 1.010, or BUN/Cr ≥ 15 combined with USG > 1.010 (dehydration status) was chosen as dehydration marker. The results found that the combination index may be superior to either of them as dehydration marker

Between December 2014 and September 2016, 375 thrombolysed patients with ischemic stroke were enrolled in the present study. 81 patients were excluded due to different reasons: 68 patients with more than 4.5 hr between the onset of neurological symptoms and rt‐PA treatment and 13 patients lack of complete clinical data. A total of 294 patients within 4.5 hr of onset were consistent with inclusion and exclusion criteria (Table 1). In the present study, BUN/Cr ≥ 15, USG > 1.010, or BUN/Cr ≥ 15 combined with USG > 1.010 (dehydration status) was chosen as dehydration marker. The results found that the combination index may be superior to either of them as dehydration marker, as shown in Figure 1.

**Table 1:** Clinical and demographic characteristics

Variables	mRS 0–2 (*n *= 191)	mRS 3–6 (*n *= 103)	*p* value
Age 65 or older, *n* (%)	70 (36.6)	54 (52.4)	.006
Male, *n* (%)	142 (74.3)	76 (73.8)	.511
WBC (10^9^/L) at admission	8.86 ± 3.41	9.07 ± 3.47	.459
RBC (10^12^/L) at admission	4.64 ± 0.50	4.64 ± 0.45	.843
Fibrinogen (g/L) at admission	2.85 ± 0.80	3.14 ± 1.08	.049
D‐Dimer (mg/L) at admission	1.26 ± 2.99	1.79 ± 2.57	.006
Blood glucose (mmol/L)	7.54 ± 3.38	8.92 ± 4.00	<.001
BUN at admission (mg/dl)	5.86 ± 1.83	6.13 ± 1.82	.282
Cr at admission (mg/dl)	71.00 ± 21.66	67.64 ± 17.76	.168
BUN/Cr at admission	18.20 ± 5.73	19.89 ± 6.10	.020
USG at admission	1.01 ± 0.01	1.02 ± 0.01	.002
Systolic blood pressure (mmHg)	152.31 ± 20.85	161.72 ± 22.33	.003
Diastolic blood pressure (mmHg)	87.37 ± 12.37	90.1 ± 12.02	.06
Dehydration status, *n* (%)	77 (40.3)	68 (66.0)	.002
Hyperhomocysteinemia, *n* (%)	76 (39.8)	39 (37.9)	.942
NIHSS at admission	6.98 ± 5.65	13.45 ± 8.11	<.001
Diabetes mellitus, *n* (%)	37 (19.4)	20 (19.4)	.554
Hypertension, *n* (%)	99 (51.8)	63 (61.2)	.079
Myocardial infarction, *n* (%)	19 (9.9)	7 (6.8)	.248
Cerebral infarction, *n* (%)	39 (20.4)	25 (24.3)	.267
Smoking, *n* (%)	88 (46.1)	38 (36.9)	.081
Hyperlipidemia, *n* (%)	108 (58.1)	46 (45.5)	.028
Blood glucose (mmol/L)			
≤8	134 (72.0)	58 (56.3)	.007
>8	52 (28.0)	45 (43.7)	
Systolic pressure (mmHg)			
≤150	176 (93.1)	87 (84.5)	.018
>150	13 (6.9)	16 (15.5)	
USG at admission			
≤1.010	75 (39.3)	28 (27.2)	.025
>1.010	116 (60.7)	75 (72.8)	
BUN/Cr at admission			
< 15	125 (65.4)	87 (84.5)	.001
≥15	66 (34.6)	16 (15.5)	
TOAST classification, *N* (%)			
Large artery atherosclerosis	134 (70.2)	83 (80.6)	.001
Small artery occlusion	36 (18.8)	5 (4.9)	
Cardioembolism	10 (5.2)	14 (13.6)	
Undetermined	11 (5.8)	1 (1.0)	

WBC, white blood cell; RBC, red blood cell; BUN, blood urea nitrogen; NIHSS, National Institutes of Health stroke scale; TOAST, trial of org 10172 in acute stroke treatment.

Continuous data expressed as mean ± SD.

According to the mRS 90 days after onset, 294 eligible patients with acute ischemic stroke after thrombolysis were divided into two groups. Table 1 summarizes the clinical and demographic characteristics of the patients in this study. Compared with mRS 0–2 patients, the patients with mRS 3–6 had older age onset, higher fibrinogen, higher ratio of USG > 1.010, higher blood glucose, higher BUN/Cr, higher D‐dimer, higher NHISS score at admission, and higher ratio of dehydration status. Of past medical history, hyperlipidemia has significant relationship with mRS 3–6. BUN/Cr (≥15 vs. <15), NIHSS (≥6 vs. ≤5), blood glucose (≥8 vs. <8), and SBP (>150 vs. ≤150) at admission were stratificated for further analysis. The results showed that BUN/Cr ≥ 15, NIHSS ≥ 6, blood glucose ≥8, and SBP > 150 were markedly associated with mRS 3–6. Furthermore, a significant difference (*p* < .05) was found among TOAST classification in two groups: there existed more stroke patients with large artery atherosclerosis in mRS 3–6, while there existed more patients with small artery occlusion in mRS 0–2.

To evaluate the risk factors potentially associated with the mRS 3–6, we performed multivariate logistic regression adjusting for age, fibrinogen, USG, D‐Dimer, dehydration status, NIHSS, blood glucose, SBP, hyperlipidemia, and BUN/Cr at admission (Table 2). The results showed that dehydration status, higher NHISS, higher blood glucose, and higher SBP at admission were independent risk factors for predicting the long‐term poor prognosis of thromlolysed patients. Patients with a higher NHISS score ≥6 at admission who were 1.151 times more likely to develop poor prognosis compared to those NHISS score ≤5 (*p* < .001). Patients with dehydration status at admission were 2.355 times more likely to develop poor prognosis as compared to patients without dehydration (*p *=* *.013). Patients with higher blood glucose at admission were 3.622 times likely to develop the poor prognosis compared to patients with lower blood glucose (*p *=* *.009). Additionally, patients with higher SBP at admission were 2.432 times likely to develop the poor prognosis compared to patients with lower blood glucose (*p *=* *.006).

**Table 2:** Multivariate logistic regression analysis for possible factors associated with poor outcomes

Variables	β	SE	Wald χ^2^	Exp (β)	95% CI for Exp (β)	*p* value
High blood pressure	1.287	0.491	6.866	3.622	1.383–9.487	.009
High SBP	0.889	0.326	7.450	2.432	1.285–4.063	.006
Dehydration status	0.857	0.345	6.174	2.355	1.198–4.428	.013
High NIHSS at admission	0.140	0.025	31.267	1.151	1.095–1.209	<.001
Constant	−1.318	14.248	0.009	0.268		.926

β, regression coefficient; SE, standard error for regression coefficient; Exp (β), odds ratio; 95% CI for Exp (β), 95% confidence interval for Exp (β).

Based on the multivariate logistic regression analysis results, the interaction between dehydration status and severity degree of neurological deficit at the admission was further determined. The patients were subdivided into (1) neurological deficit group: NIHSS ≤ 5 (minor stroke) versus NIHSS ≥ 6 and (2) dehydration status group: dehydration status versus no dehydration status. The results showed that dehydration status was significantly associated with mRS 3–6 in thrombolysed patients with NIHSS ≥ 6, but not NHISS score ≤5 (Table 3). It is interesting to note that 38.2% of the patients in the mRS 0–2 group were also dehydrated. The results imply that the dehydration was not the only factor of poor outcome and multiple factors act together to contribute to the poor outcome in the thrombolysed patients.

**Table 3:** Effect of dehydration on prognosis of patients with stroke

	NIHSS ≤ 5		*p* value	NIHSS ≥ 6		*p* value
	mRS 0–2	mRS 3–6		mRS 0–2	mRS 3–6	
No dehydration status	68 (61.8)	9 (40.9)	.146	43 (54.4)	29 (35.4)	.006
Dehydration status	42 (38.2)	12 (57.1)		36 (45.6)	53 (64.6)	

To investigate the relationship of the severity degree of dehydration status with long‐term prognosis in thrombolysed patients, the patients were further subdivided into two groups: 15 ≤ BUN/Cr < 20 plus 1.010 < USG < 1.020 and BUN/Cr ≥ 20 plus USG ≥ 1.020, based on BUN/Cr value combined with USG > 1.010. The results further showed that the degree of dehydration status had significant relationship with long‐term prognosis in thrombolysed patients (Table 4).

**Table 4:** Effect of dehydration degree on prognosis of patients with stroke

Dehydration status	mRS 0–2	mRS 3–6	*p* value
15 ≤ BUN/Cr < 20 plus 1.010 < USG < 1.020	30 (69.8)	18 (40.9)	.006
BUN/Cr ≥ 20 plus USG ≥ 1.020	13 (30.2)	26 (59.1)	

## 4 DISCUSSION

Although thrombolytic therapy is an effective and safe treatment for acute ischemic stroke, there are still many patients who cannot improve neurological function after thrombolysis therapy. There are many factors related with poor neurological function prognosis such as NHISS score at admission, the time of onset to admission, the area of the infarct (Bhatia et al., 2015; Ribo et al., 2016; Smith et al., 2010). So far, it is not clear whether dehydration status is associated with the prognosis of acute ischemic stroke patients with thrombolytic treatment. The present results for the first time provided the clear evidence that BUN/Cr ≥ 15 plus USG > 1.010 as a marker of dehydration may be a strong and superselective predictor of long‐term outcome of patients with acute ischemic stroke after thrombolysis therapy.

Dehydration, as indicated by an increased BUN/Cr ratio or USG (Aronson et al., 2008; Vullo‐Navich et al., 1998; Weinberg et al., 1994), is known to be relatively common among patients who have experienced stroke (Rowat, Graham, & Dennis, 2012). Some studies have reported the possible relationship of dehydration with poor neurological function outcome of acute ischemic stroke patients. For example, BUN/Cr ratio as a marker of dehydration status was an independent predictor of early neurological deterioration among patients who had suffered acute ischemic stroke (Aronson et al., 2008; Lin et al., 2011; Vullo‐Navich et al., 1998; Weinberg et al., 1994). A correlation between BUN/Cr ratio ≥15 and worse outcome was also found in stroke patients (Schrock et al., 2012). USG was also reported to be useful as a predictor of early deterioration in patients with acute ischemic stroke (Lin et al., 2011). In the present study, we found that the mRS 3–6 was associated with a higher BUN/Cr, USG, and dehydration status, compared to the mRS 0–2. However, multivariate logistic regression analysis showed that BUN/Cr ratio ≥15 combined with USG > 1.010, but not either of them was independently associated with the poor outcome. The results are different from the previous results that BUN/Cr ratio ≥15 or USG > 1.010 was an independent risk of early neurological deterioration in patients with acute ischemic stroke (Aronson et al., 2008; Lin et al., 2011, 2011; Vullo‐Navich et al., 1998; Weinberg et al., 1994). The reason may be: (1) the effect of dehydration on long‐term prognosis was determined in the present study, while the effect on short‐term prognosis in other studies; (2) thrombolysed patients with acute cerebral infarction are subjects in the present study, who are different from patients with acute cerebral infarction in other studies; (3) BUN/Cr ≥ 15 combined with USG > 1.010 was used as a marker of dehydration status in our study, while either of them as a marker in other studies; (4) mRS 0–2 was used as a good outcome in the present study, while mRS ≤ 1 was defined as a good outcome in other studies (Cappellari et al., 2013; Song et al., 2017; Tsivgoulis et al., 2016). Different mRS cut‐offs were used as good outcome in previous study, for example, mRS ≤ 1 in some studies (Cappellari et al., 2013; Song et al., 2017; Tsivgoulis et al., 2016) and mRS 0–2 in other studies (Chen et al., 2016; Neidert et al., 2016).

As discussed above, BUN/Cr ≥ 15 or USG > 1.010 was often used as a maker of dehydration status in previous studies (Akimoto et al., 2011; Bhatia et al., 2015; Lin et al., 2011; Schrock et al., 2012). USG > 1.030 has also been reported as a marker in only one study with subjects in an ultralow‐humidity environment (Su et al., 2006). In the present study, we found that BUN/Cr ≥ 15 plus USG > 1.010 as a marker of dehydration status was an independent risk factor for predicting the long‐term poor prognosis of thrombolysed patients. Furthermore, dehydration degree stratificated by BUN/Cr plus USG (but not either of them) was found to have obvious relationship with long‐term poor prognosis: obvious dehydration status (BUN/Cr ratio ≥ 20 plus USG ≥ 1.020) was attributed to poor outcome. It is not clear as to which parameters is best to investigate the relationship of dehydration status to ischemic stroke. Based on the current results, we argue that BUN/Cr ≥ 15 plus USG > 1.010 may be superior to BUN/Cr ≥ 15 or USG > 1.010 in predicting the relationship of dehydration with poor outcome in ischemic stroke, which deserved to be determined in further clinical trials.

It makes physiologic sense that dehydration causes a contraction of total plasma volume and decreased cardiac output, thus resulting in decreased blood viscosity, decreased blood pressure, impaired collateral flow and reduced cerebral perfusion (Frey et al., 1994; Schrock et al., 2012; Yamaguchi, Minematsu, & Hasegawa, 1997). Finally, these changes may disrupt the recanalization of the occlusion artery by rt‐PA. It is reasonable to refer that quickly hydrating may increase the recanalization rate with resulting improved outcome. The proposal deserved to be determined in further clinical trials. If it is true, the BUN/Cr ratio and USG should also be tested as soon as possible for thrombolysed stroke patients.

In the present study, NIHSS score at admission was also found to be an independent risk factor for predicting the poor prognosis of thrombolysed patients with acute ischemic stroke. In agreement with our results, high NIHSS score at admission were also found to play a role in poor outcome in thrombolysed patients with ischemic stroke (Ryu et al., 2010; Smith et al., 2010; Zhang et al., 2010). Furthermore, we observed that severity degree of neurological deficit at admission was associated with the prognosis in the dehydrated patients. Dehydration status was obviously associated with long‐term poor prognosis in thrombolysed patients with NIHSS ≥ 6, but not NHISS score ≤5. Namely, the more severe dehydration status at admission, the worse the prognosis. Additionally, high blood glucose levels was also found to be an independent risk factor of prognosis of thrombolysed patients with acute ischemic stroke in our study, which was consistent with previous reports (Bruno & Hamilton, 2001; Bruno et al., 1999; Demchuk et al., 1999; Kase et al., 2001). Higher blood glucose levels may lead to secondary tissue acidosis and oxygen‐derived free radicals increase, resulting in brain cell metabolism disorders, increased brain tissue damage, and the blood–brain barrier disruption (Godoy, Behrouz, & Di Napoli, 2016; Tureyen et al., 2011). Furthermore, the study shows that higher SBP levels before thrombolysis may be detrimental to poor outcome. The effects of blood pressure at admission on the prognosis of patients with acute ischemic stroke are complex and controversy. Both high and low SBP were independently associated with increased early death and late death or dependency (Leonardi‐Bee et al., 2002; Sprigg et al., 2006). The clinical meaning of the findings is not clear and worthy of attention.

The present study has some limitations. First, this was a retrospective cohort study. Second, this was a small sample, single‐center study. Thus, a prospective, big sample, multicenter clinical trial should be designed. Third, the BUN/Cr ratio ≥ 15 plus USG > 1.010 as dehydration marker may be superior to either of them in thrombolysed patients, which deserved to be further confirmed in clinical trials with big sample. It is not clear whether the combination index is suitable for other subjects.

In conclusion, the present findings found that BUN/Cr ≥ 15 combined with USG > 1.010 as marker of dehydration status was an independent risk factor for long‐term poor prognosis of thrombolysed patients with acute ischemic stroke, suggesting that rapidly correcting dehydration may be one of therapy strategy in the thrombolysed patients with acute ischemic stroke.

## DISCLOSURES

None.
